# Thyroid abnormality in patients with psoriasis: prevalence and association with severity^☆^

**DOI:** 10.1016/j.abd.2022.12.007

**Published:** 2023-08-17

**Authors:** Luiza de Castro Fernandes, Ana Carolina Belini Bazan Arruda, Lisa Gava Baeninger, Debora Pedroso Almeida, Danilo Villagelin

**Affiliations:** a Department of Internal Medicine, Universidade Estadual de Campinas, Campinas, SP, Brazil; b Department of Dermatology, Hospital da Pontifícia Universidade Católica de Campinas, Campinas, SP, Brazil; c Department of Endocrinology and Metabology, Hospital da Pontifícia Universidade Católica de Campinas, Campinas, SP, Brazil

**Keywords:** Arthritis, psoriatic, Hypothyroidism, Psoriasis

## Abstract

**Background:**

Psoriasis is associated with several comorbidities and its association with thyroid abnormality has been hypothesized.

**Objective:**

To assess the prevalence of thyroid abnormality in Brazilian patients with psoriasis and to analyze its association with severity, presence of psoriatic arthritis and immunobiological treatment. Additionally, to compare results with literature as a control.

**Methods:**

In this observational study, clinical and laboratory data of patients followed from January 2018 to December 2019 were analyzed. Thyroid abnormality was assessed through the current history of thyroid disease and laboratory tests - thyrotropin (TSH), free thyroxine (FT4), antithyroid peroxidase (anti-TPO) and antithyroglobulin (anti-TG) antibodies. Patients were classified according to psoriasis severity - Psoriasis Area and Severity Index (PASI), presence of psoriatic arthritis, and current treatment. Subsequently, the results were compared with a control group selected from the literature review.

**Results:**

Of the 250 included patients, 161 were eligible. The prevalence of thyroid abnormality was 28.57% and of hypothyroidism, 14.91%. The mean age was 55 years and the median PASI was 2.2. There was no association between thyroid abnormality and PASI (p = 0.8), presence of psoriatic arthritis (p = 0.87), or use of immunobiological therapy (p = 0.13). The literature control group included 6,227 patients and there was a statistically significant difference for the hypothyroidism variable (p < 0.0001).

**Study limitations:**

Absence of a control group from the same center.

**Conclusion:**

This was one of the first Brazilian studies on the prevalence of thyroid abnormality in patients with psoriasis.

## Introduction

The estimated prevalence of psoriasis in Brazil is 1.3%, with higher frequencies in the Southern and Southeastern regions.^1^ The association with a wide range of comorbidities is recognized, particularly psoriatic arthritis (PA), metabolic syndrome, cardiovascular disease, diabetes mellitus, psychiatric disorders, asthma, and inflammatory bowel disease.^2^, ^3^ Immune-mediated or autoimmune diseases such as rheumatoid arthritis, celiac disease, and vitiligo are also well-known associations.^4^

Although the association between psoriasis and thyroid abnormality has been investigated by several authors (Table 1), case-control and population-based studies have been published without definitive conclusions.^5^, ^6^, ^7^, ^8^, ^9^, ^10^, ^11^, ^12^ The correlation with psoriasis severity^13^, ^14^ and the frequency (incidence or prevalence) of thyroid abnormality in patients with psoriatic disease^15^, ^16^, ^17^, ^18^, ^19^ have also been previously investigated.

**Table 1:** Psoriasis and thyroid abnormality – previous studies.

Authors/Year of publication	Study design	Objective	n (sample size)	Country	Thyroid markers	Findings
Arican, Bilgic and Koc, 2004^a^	Case-control	Comparison of serum thyroid hormone levels	103 patients × 96 controls	Turkey	T3T, T3L, T4T, T4L, TSH	Elevated FT4 and FT3 in patients with psoriasis (p < 0.05), elevated PASI in these patients (p < 0.001)
Antonelli et al. 2006	Case-control	Prevalence of thyroid disorders in patients with PA vs. controls	Patients: 80 PA/ 112 rheumatoid arthritis / 400 controls	Italy	T3L, T4L, TSH, anti-TPO, anti-TG, thyroid ultrasonography	Autoimmune thyroid dysfunction (patients × controls): 33%×18% in females and 25%×5% in males (p = 0.0001)
Gul et al. 2009^a^	Case-control	Association of autoimmune thyroid diseases with psoriasis	105 patients × 96 controls	Turkey	T3L, T4L, TSH, anti-TPO, anti-TG, thyroid ultrasonography	No difference between the groups
James, Hill and Feldman 2016	Cross-sectional	Prevalence of hypothyroidism in patients with rosacea or psoriasis compared to other dermatological diseases	Human Database – 1,667,943 patients	USA	CID-10	Prevalence of hypothyroidism of 17.5% in patients with psoriasis. Frequency of hypothyroidism did not differ from other dermatological patients
Lai and Yew 2016	Cross-sectional	Association of psoriasis and thyroid disease		India	Thyroid hormones and autoantibodies	Low levels of TSH in patients with psoriasis (p < 0.019)
Fallahi et al. 2017	Case-control	Incidence of clinical or subclinical thyroid dysfunction in patients with PA	97 patients × 97 controls	Italy	T4L, T3L, TSH, anti-TPO, anti-TG, thyroid ultrasonography	High incidence of anti-TPO positivity (p = 0.017), hypothyroidism (p = 0.017), thyroid dysfunction (p = 0.002), hypoechogenicity (p = 0.009) and thyroid autoimmunity (p = 0.007)
Khan et al. 2017	Cross-sectional analysis of a cohort	Association of positive anti-TPO, TSH and FT4 with psoriatic disease	Population – part of the Rotterdam Study – 8,214 participants	The Netherlands	T4L, TSH, anti-TPO	No association considering incident or prevalent psoriatic disease
Kiguradze et al. 2017	Cross-sectional	Association between psoriasis and HT	Population – Northwestern Medicine Enterprise Data Warehouse – 856,615 participants – 9,654 diagnoses of psoriasis – 1,745 diagnoses of HT	USA	T3L, TSH, anti-TPO, anti-TG	Positive association (OR = 2.49 [95% CI 1.79–3.48] p < 0.0001)
Vassilatou et al. 2017^a^	Case-control	Prevalence of autoimmune thyroiditis in patients with psoriasis	114 patients with psoriasis × 286 controls	Greece	T3T, T4T, T4L, TSH, anti-TPO, anti-TG	No difference in prevalence
Valejo, Coelho and Brasileiro 2018	Cross-sectional	Prevalence of thyroid dysfunction	55 patients with psoriasis	Portugal	T3L, T4L, TSH, anti-TPO, anti-TG	Prevalence of 9.1%
Alidrisi et al. 2019^a^	Case-control	Prevalence of HT in patients with psoriasis compared to controls	56 patients × 54 controls	Iraq	T4L, TSH, anti-TPO, anti-TG, thyroid ultrasonography	Higher prevalence of anti-TPO (25% × 9.3% p = 0.02), anti-TG (30.4% × 11.1%; p = 0.01), hypoechogenicity (30.4%×9.3%; p = 0.02), pseudonodularity (16.1% × 0%; p = 0.002) and elevated vascularity on ultrasonography in patients with psoriasis (35.7% × 5.6%; p = 0.001)
Hansen et al. 2019^a^	Case-control	To assess thyroid function	Population – Danish General Population Study – 1,127 patients × 5,637 controls	Denmark	T3T, T4L, TSH, anti-TPO	Elevated T3T levels in patients with psoriasis (1,72 × 1.69; p = 0.01)
Mallick 2019	Cross-sectional	Frequency of thyroid disorders in patients with psoriasis	112 patients	Pakistan	T3L, T4T, TSH	15.2%
Wang et al. 2019	Cohort	Risk of thyroid disease in patients with psoriasis	Population – Taiwan National Health Insurance Research Database – 13,266 patients with PA/ 149,576 with isolated psoriasis/ 162,842 controls	Taiwan	CID-10	High incidence of hyperthyroidism in patients with psoriasis (aHR = 1.22; 95% IC 1.11–1.33), Graves disease (aHR = 1.26; 95% IC 1.13–1.41), incidence of hypothyroidism (aHR = 1.38; 95% IC 1.23–1.56) and HT (aHR = 1.47; 95% CI 1.18–1.82)
Namiki et al. 2020	Cross-sectional	Prevalence of thyroid dysfunction	85 patients: 51 with psoriasis vulgaris/ 23 PA/ 11 GPP	Japan	T3L, T4L, TSH	Prevalence of 8%, 13% and 45%, respectively
Wu et al. 2021	Cross-sectional	Association of psoriasis with increased risk of thyroid disease	National Health and Nutrition Examination Survey – 15,091 patients	USA	Self-reported disease	Positive association (OR = 1.61 [95% CI 1.01–2.55] p = 0.043)
Valduga et al. 2021	Cross-sectional	Prevalence of HT	60 patients × 60 controls	Brazil	TSH, T4L, anti-TPO, anti-TG	Prevalence of HT (OR = 3.8 [95% CI 1.18–12.6] p = 0.03)

a Studies included for literature control.

Considering the importance of the association for clinical management and the scarcity of these data in the Brazilian population, this study aimed to evaluate the prevalence of thyroid abnormality in patients with psoriasis and to analyze its association with three factors: severity (measured by the Psoriasis Area and Severity Index – PASI); the presence of psoriatic arthritis; and treatment with immunobiologicals. Additionally, the study assessed publications of interest, identifying the study design and inclusion of a control group in the studies, and according to the adopted criteria, compared by meta-analysis the group of cases in this study with the control group from the selected publications.

## Methods

After approval by the local Research Ethics Committee, a cross-sectional observational study was conducted in the Dermatology department. Data from 250 patients were analyzed.

All patients followed at the Dermatology Outpatient Clinic of Psoriasis and Immunobiologicals from January 2018 to December 2019 were considered eligible, constituting a convenience sample. The inclusion criteria were: diagnosis of psoriasis vulgaris and duration of treatment – considering the use of topical treatments, phototherapy, conventional systemic drugs (acitretin, methotrexate and cyclosporine), or immunobiological agents (infliximab, etanercept, adalimumab, ustekinumab, secukinumab). The exclusion criteria were: diagnosis of other forms of psoriasis other than vulgaris, patients who did not agree to participate in the study, those younger than 12 years, previous thyroidectomy, current treatment with medications that could affect thyroid function (lithium, amiodarone, anticonvulsants and interferon) and absence of records in the medical file regarding thyroid hormones or antibodies.

### Study design

The clinical data collected from the medical records were: age, sex, height, weight, body mass index (BMI), presence of psoriatic arthritis (and affected joints), disease duration (months), previous and current treatment for psoriasis, duration of current treatment (months), current PASI, presence of comorbidities (hypertension, diabetes, depression or anxiety, dyslipidemia, smoking, alcoholism, non-alcoholic liver disease, osteoarthritis, anterior uveitis), previous thyroid diseases (hypothyroidism, hyperthyroidism, nodule), previous autoimmune diseases (systemic lupus erythematosus, type I diabetes, vitiligo, rheumatoid arthritis, Sjögren's syndrome) and medications used.

The laboratory data collected were: serum thyrotropin (TSH), free thyroxine (FT4), anti-thyroid peroxidase (anti-TPO) antibodies, antithyroglobulin (anti-TG) antibodies, fasting glucose, total cholesterol and fractions, and triglycerides.

Physical examination and data collection were supervised by trained dermatologists. All patients were informed about the study and signed the informed consent form.

### Clinical evaluation

Psoriasis severity was clinically classified according to PASI. Trained dermatologists calculated the index during follow-up visits. As it is already well established, the score, ranging from 0 to 72, allows the division of patients into two groups: mild psoriasis (PASI ≤ 10) and moderate/severe psoriasis (PASI > 10).^20^ The groups were compared regarding the prevalence of thyroid abnormalities.

The frequency of the variable was also compared considering the current treatment (immunobiological vs. non-immunobiological therapy) and the coexistence of PA (present vs. absent). The rheumatological diagnosis of PA, in turn, was based on clinical and laboratory criteria.

### Thyroid abnormality

The prevalence of thyroid abnormality was defined as the presence of one of the following aspects: previous diagnosis of hypothyroidism, antibody positivity (anti-TPO or anti-TG), altered serum TSH values (<0.27 mIU/L or >4.5 mIU/L).

### Laboratory methods

Serum TSH, FT4, anti-TPO and anti-TG values were measured by chemiluminescence assays. The reference values were respectively: 0.27–4.5 mIU/L, 0.93–1.7 ng/dL, positive >34 IU/mL and >115 IU/mL. Fasting blood glucose was estimated using the hexokinase method and serum cholesterol and triglyceride levels were assessed using the enzymatic colorimetric method.

### Literature control group

The literature review included Pubmed, Embase and Scopus databases, limited to studies with human subjects and publication in English or Portuguese. The selection period comprised January 2002 to May 31, 2022.

The descriptors used were: ((Psoriasis [title]) AND (thyroid [title]) OR (hypothyroidism [title]) OR (thyroiditis [title]) OR (Hashimoto thyroiditis [title])) e ((Psoriatic [title]) AND (thyroid [title]) OR (hypothyroidism [title]) OR (thyroiditis [title]) OR (Hashimoto thyroiditis[title])). Articles from other sources were not considered.

The available title and abstract were used as selection criteria, after excluding duplicate articles. The studies conducted with the objective of evaluating the frequency or association of autoimmune thyroid disease and psoriatic disease were considered eligible.

The inclusion criteria were: cross-sectional, case-control, or cohort study designs, and the presence of a control group in the study design. The exclusion criteria comprised studies with a case group consisting exclusively of patients with psoriatic arthritis; and those who used only the ICD (International Classification of Diseases) without laboratory data for the diagnosis of hypothyroidism in the publication.

Data collected from selected studies with a control group included: authors names, year of publication, number of patients belonging to the control group, number of patients diagnosed with hypothyroidism, number of patients with positive anti-TPO, positive anti-TG, and altered TSH, or mean and standard deviation of TSH.

The information obtained through literature control was grouped and evaluated according to a meta-analysis so that the data could be weighted, aiming to integrate the results of the studies.

### Statistical analysis

The SAS System for Windows v9.4 (SAS Institute Inc. Cary, NC, USA) was used for the statistical analysis, and p values ≤ 0.05 were considered significant.

The sample profile was defined by calculating the frequency of categorical variables in absolute numbers (n) and percentages (%). Descriptive measures (mean, standard deviation, minimum/maximum values and median) were used for quantitative variables.

The Chi-square or Fisher exact test was used for the analysis of the correlation between baseline variables and characteristics and the existence of comorbidities, for categorical variables, and the Mann-Whitney test for numerical variables.

For the analysis of the literature control group, a meta-analysis was applied to estimate the proportion or mean and its respective confidence interval using the random model via linear models. To compare the group of cases with the control group in the literature, the chi-square test was used for proportions and Student *t* test was applied to compare continuous measurements between the two groups.

## Results

Initially, 250 patients were considered eligible, but 15 were not included because they had a diagnosis of exclusive palmoplantar psoriasis or generalized pustular psoriasis. Of the total of 235 patients included in the study, 74 were excluded (64 due to incomplete information in the medical records, three due to the use of medications that alter thyroid function and seven due to loss of follow-up). Therefore, the final sample consisted of 161 patients, whose data were analyzed.

Among the sample patients, 64.60% were male, with a mean age of 55 years. The prevalence of thyroid abnormality was 28.57% and of hypothyroidism, 14.91%. The following autoimmune diseases were detected in the sample: Sjögren's syndrome, vitiligo and type I diabetes (in one, two and one patients, respectively). With regard to treatment, 44.09% of the patients were using immunobiological therapy, 37.88% were using non-immunobiological systemic treatment, 3.72% were using phototherapy and 14.28% were using exclusive topical therapy. Considering the PASI, 15.55% (21 patients) were considered to have moderate/severe disease (Tables 2 and 3).

**Table 2:** Sample overall frequencies.

Clinical characteristics	Frequency (number of patients)	Percentage (%)
Male	104	64.6
Psoriatic arthritis	54	33.54
Diabetes	45	27.95
Hypertension	70	43.48
Depression/anxiety	10	6.21
Dyslipidemia	52	32.3
Autoimmunity (except hypothyroidism)	4	2.48
Thyroid abnormality	46	28.57
Hypothyroidism	24	14.91
Altered TSH	14	10.29
Positive anti-TPO	13	8.55
Positive anti-TG	7	4.83

**Table 3:** Descriptive measures of quantitative variables.

Variable	Mean	Minimum‒Maximum	Median	Standard deviation
Age	55.17	12‒94	56	16.10
Weight	78.78	44‒125	77	15.58
Height	1.67	1.48‒1.96	1.67	0.09
BMI	28.20	18.80‒47.05	27.7	5.12
Disease duration (months)	215.22	12‒720	192	152.25
PASI	5.62	0‒57	2.2	8.13

Quantitative variables (age, BMI, disease duration and PASI) were compared with gender and the presence or absence of comorbidities (hypertension, dyslipidemia, diabetes, hypothyroidism and PA). There was no difference in PA frequency between the male and female sex. Hypertension, dyslipidemia and hypothyroidism were more prevalent at older ages (OR = 1.066 [95% CI 1.038–1.094] p < 0.0001; OR = 1.039 [95% CI 1.014–1.064] p = 0.0025 and OR = 1.048 [95% CI 1.009–1.077] p = 0.0125, respectively). In parallel, diabetes was more frequent in older patients (OR = 1.048 [95% CI 1.020–1.076] p = 0.0006) and those with higher BMI (OR = 1.083 [95% CI 1.007–1.164] p = 0, 0314). No statistically significant difference was found considering hypothyroidism and PASI.

Patients allocated considering PASI (mild or moderate/severe), PA (presence or absence) and current treatment (immunobiologicals or non-immunobiologicals) were compared regarding thyroid abnormality. As depicted in Tables 4, 5 and 6, no statistically significant difference was found between these groups.

**Table 4:** Thyroid abnormality and PASI (Psoriasis Area and Severity Index).

PASI	Moderate/severe (> 10) n (%)	Mild (≤ 10) n (%)	p-value
Variables			
Hypothyroidism+	4 (2.96)	15 (11.11)	0.49
Hypothyroidism−	17 (12.59)	99 (73.33)	
Altered TSH	0 (0)	10 (8.70)	0.35
Normal TSH	17 (14.78)	88 (76.52)	
Anti-TPO+	3 (2.29)	8 (6.11)	0.38
Anti-TPO−	18 (13.74)	102 (77.86)	
Anti-TG +	1 (0.79)	4 (3.17)	0.58
Anti-TG−	19 (15.08)	102 (80.95)	
Thyroid abnormality+	5 (3.70)	30 (22.22)	0.80
Thyroid abnormality−	16 (11.85)	84 (62.22)

**Table 5:** Thyroid abnormality and psoriatic arthritis.

Psoriatic arthritis	Presence n (%)	Absence n (%)	Valor p
Variable			
Hypothyroidism+	9 (5.59)	15 (9.32)	0.65
Hypothyroidism−	45 (27.95)	92 (57.14)	
Altered TSH	3 (2.21)	11 (8.09)	0.38
Normal TSH	42 (30.88)	80 (58.82)	
Anti-TPO+	5 (3.29)	8 (5.26)	0.76
Anti-TPO−	46 (30.26)	93 (61.18)	
Anti-TG+	1 (0.69)	6 (4.14)	0.42
Anti-TG−	48 (33.10)	90 (62.07)	
Thyroid abnormality+	15 (9.32)	31 (19.25)	0.87
Thyroid abnormality−	39 (24.22)	76 (47.20)

**Table 6:** Thyroid abnormality and immunobiological therapy.

Current treatment	Immunobiological	Non-immunobiological	p-value
Variable	n (%)	n (%)	
Hypothyroidism+	9 (5.59)	15 (9.32)	0.48
Hypothyroidism−	62 (38.51)	75 (46.58)	
Altered TSH	4 (2.94)	10 (7.35)	0.17
Normal TSH	58 (42.65)	64 (47.06)	
Anti-TPO+	5 (3.29)	8 (5.26)	0.56
Anti-TPO−	65 (42.76)	74 (48.68)	
Anti-TG+	3 (2.07)	4 (2.76)	1.0
Anti-TG−	65 (44.83)	73 (50.34)	
Thyroid abnormality+	16 (9.94)	30 (18.63)	0.13
Thyroid abnormality−	55 (34.16)	60 (37.27)	

Patients on non-immunobiological therapy were divided into groups according to the type of treatment: exclusively topical, phototherapy and systemic medication. There was, however, no statistically significant difference between the groups for the hypothyroidism (p = 0.10) and thyroid abnormality (p = 0.17) variables.

### Control group

In the literature review, 80 articles were found in the Pubmed database, 90 in the Embase database and 85 in the Scopus database. Of the articles of interest, 24 duplicates and one article written in the Hebrew language were not considered. A total of 17 articles were identified as eligible. Subsequently, two were excluded for reporting a group of cases consisting exclusively of patients with psoriatic arthritis, four due to lack of a control group, two for exclusively using the ICD for diagnosis and three for insufficient laboratory data in the body of the article. Therefore, a total of six articles were sent for statistical analysis, all with a cross-sectional or case-control design (Table 1).^5^, ^6^, ^11^, ^13^^,^^14^, ^21^

The control sample size corresponded to 6,227 patients. The sample evaluated for each of the variables differed according to the data studied in each article. Therefore, the total number of patients included for hypothyroidism was 6,035 (four articles),^5^, ^11^, ^13^, ^21^ anti-TPO, 6,069 (four articles),^5^, ^6^, ^11^, ^13^ anti-TG, 443 (three articles),^5^, ^6^, ^13^ altered TSH, 96 (one article),^6^ and mean TSH, 6,071 (four articles).^5^, ^11^, ^13^, ^14^

The comparison between the results of cases and controls in the literature for the variables hypothyroidism, positive anti-TPO, positive anti-TG and altered TSH showed a statistically significant difference for hypothyroidism. While in the case group, the proportion of hypothyroidism was 14.91%, in the control group it was 3.58% (random effects model: 0.035 [95% CI 0.016‒0.074]; p < 0.0001).

Furthermore, the mean TSH in the case group was 2.67 mIU/L, while the weighted mean of TSH in the control group was 2.06 mIU/L, with a statistically significant difference between the groups (p = 0.026).

## Discussion

The association between psoriasis and several comorbidities has been an important topic of research and current evidence suggests a multifactorial aspect of the disease, expanding its spectrum and its impact beyond the dermatological and rheumatological areas. Therefore, knowing the possible associations with other comorbidities, such as thyroid disease, is a key element in improving care.

The activation of the inflammatory pathway by tumor necrosis factor-alpha, interleukin 23, and interleukin 17 (TNF-α, IL-23, IL-17) is involved in the pathophysiology of psoriasis.^22^ The role of this pathophysiological pathway has been studied in thyroid diseases.

Previous studies have shown that the status of epidermal proliferation changes with thyroid disease,^23^ and thyroid hormones are able to induce the production of epidermal growth factor (EGF), whose persistence could be associated with the hyperproliferative state of psoriasis.^24^, ^25^

It is currently known that psoriasis has a high frequency of Treg/IL17+ lymphocytes and recent studies have demonstrated high levels of T-helper 17 (Th17) lymphocytes, both in the peripheral tissue and in the thyroid tissue of patients with autoimmune thyroid disease, as well as Th17 expression in patients with Hashimoto thyroiditis (HT).^26^, ^27^ This pathway may be related not only to the pathophysiology of psoriasis but to that of immune-mediated thyroid diseases, suggesting a connection between them.

Cytokine CXCL10 plays an important role in Th1 lymphocyte chemoattraction and is found at high levels in patients with PA and HT when compared to patients with isolated PA,^13^, ^28^ representing another point in common in the pathophysiological pathway.

Although already hypothesized and demonstrated in previous studies,^7^, ^8^, ^18^ this is one of the first studies with Brazilian data to assess the prevalence of thyroid abnormality in patients with psoriasis vulgaris.

The prevalence of hypothyroidism of 7.4% and of chronic autoimmune thyroid disease of 16.9% in the Brazilian population has been previously estimated.^29^, ^30^ The prevalence of HT in the study by Valduga et al., which analyzed 60 Brazilian patients and 60 controls, was 21.6% for the case group.^21^ The sample evaluated herein, which had a more substantial number of participants, found prevalence rates of 28.57% for thyroid abnormality and 14.91% for hypothyroidism. Additionally, 10.29% of the patients had altered TSH levels and positive anti-TPO and anti-TG were demonstrated in 8.55% and 4.83% of the patients, respectively. However, it should be noted that the present study was conducted in a specialized outpatient clinic and, therefore, is not comparable to the general Brazilian population.

Similar results were demonstrated by other authors. Mallick et al. found a frequency of 15.2% for thyroid disorders in a sample of 112 patients with psoriasis vulgaris;^16^ Namiki et al. studied thyroid dysfunction in 85 patients and found a prevalence of 8% in patients with psoriasis without PA, 13% in patients with PA, and 45% in patients with generalized pustular psoriasis (GPP).^17^ In contrast, patients with GPP were excluded from this study, although they may be included in future evaluations.

It has been demonstrated a high frequency of thyroid autoimmunity in females, who are up to eight times more frequently affected than males.^31^ In the assessed sample, 64.6% of the patients were men, which should imply a lower frequency of thyroid abnormality, suggesting that the high frequency found should be valued and discussed in future studies.

The available systemic medications for psoriasis vulgaris are mainly reserved for patients with moderate to severe clinical conditions during the course of disease evolution. In this carried-out study, 83.84% of the patients used systemic drug therapy. However, 15.55% had PASI > 10, with mean and median values corresponding to 5.62 and 2.2 (0–57), respectively. Most patients were, therefore, undergoing adequate treatment and under disease control during the study. There was no association between PASI and thyroid abnormality. However, the score is dynamic, being impacted by treatment and changing during the follow-up, so it does not reflect disease severity when assessed at intervals.

In line with other studies, Arican et al. established an association between thyroid disease and PASI, demonstrating higher index values in patients with thyroid abnormality.^14^, ^17^ Treatment withdrawal for at least one month, as adopted by the authors, made the PASI a more reliable score for assessing disease severity.

Immunobiological therapy, when compared to conventional therapy, was not associated with thyroid abnormality (p = 0.13). Similarly, no statistically significant difference was found between patients when divided into the three groups of non-immunobiological treatment: systemic, phototherapy, and topical treatments, considering the variables of hypothyroidism (p = 0.10) and thyroid abnormality (p = 0, 17).

PA, prevalent in up to 30% of the patients, has a significant impact on quality of life and is associated with the occurrence of other comorbidities.^2^ The association with hypothyroidism, positive anti-TPO, and ultrasonographic findings (thyroid hypoechogenicity) has been previously demonstrated.^18^, ^19^ There was no association of PA in the assessed sample, considering hypothyroidism (p = 0.65), anti-TPO (p = 0.76), or thyroid abnormality (p = 0.87). Ultrasonography for thyroid assessment was not performed in this study, which constitutes a limitation.

Historical control and literature control are most frequently used in clinical trials, with benefits regarding the reduction of costs and study duration.^32^, ^33^ The literature review and use of the control group from the selected articles for comparison complement the study.

The prevalence of hypothyroidism among patients with psoriatic disease when compared to literature control data was higher, with a statistically significant difference. These data, corroborated by those observed in the Brazilian study by Valduga et al.,^21^ justifiy that greater attention be paid to thyroid function in patients with psoriasis.

However, some considerations must be made related to the study design. The composition of the control group of the articles included in the meta-analysis varied, consisting of patients with other dermatological diseases in the studies by Arican et al.^14^ and Gul et al.,^6^ while in the study by Valduga et al.^21^ it consisted of patients from the gynecology and ophthalmology clinic. Meanwhile, in the article by Hansen et al.^11^ patients belonged to the Danish General Suburban Population Study database. Therefore, the control group obtained from the literature review was not restricted to a single population, reducing the risk of choosing a control group that was very different from the cases and improving the analysis. However, the fact that the control group did not come from the same center or the same geographic area is a limiting factor.

The way hypothyroidism was diagnosed also varied between the studies. The article by Hansen et al.^11^ used self-reported hypothyroidism information, while the others made the diagnosis based on laboratory data obtained during the study. In the present study, the patients with a previous diagnosis of hypothyroidism and those who had altered laboratory data, including the diagnosis of thyroid abnormality, were included.

## Conclusion

Although many studies have investigated the association or frequency of thyroid abnormality in patients with psoriasis, this was one of the first studies carried out with Brazilian data. The prevalence of thyroid abnormality was 28.57% and that of hypothyroidism was 14.91%. Moreover, the prevalence of hypothyroidism when compared to the literature control was positive. This analysis complements the current study, allowing a more complete comparison and assessment of the data obtained in the present series.

The analyzed results allow future studies to be conducted aiming at evaluating the existence of an association or the screening for thyroid autoimmunity in this population group.

## Financial support

None declared.

## Authors' contributions

Luiza de Castro Fernandes: Design and planning of the study; data collection, or data analysis and interpretation; statistical analysis; drafting and editing of the manuscript or critical review of important intellectual content; collection, analysis, and interpretation of data; effective participation in research orientation; intellectual participation in the propaedeutic and/or therapeutic conduct of the studied cases; critical review of the literature; approval of the final version of the manuscript.

Ana Carolina Belini Bazan Arruda: Design and planning of the study; data analysis and interpretation; drafting and editing of the manuscript or critical review of important intellectual content; collection, analysis, and interpretation of data; effective participation in research orientation; intellectual participation in the propaedeutic and/or therapeutic conduct of the studied cases; critical review of the literature; approval of the final version of the manuscript.

Lisa Gava Baeninger: Design and planning of the study; data collection, drafting and editing of the manuscript; collection, analysis, and interpretation of data; effective participation in research orientation; critical review of the literature; approval of the final version of the manuscript.

Debora Pedroso Almeida: Design and planning of the study; data collection, drafting and editing of the manuscript; collection, analysis, and interpretation of data; effective participation in research orientation; critical review of the literature; approval of the final version of the manuscript.

Danilo Villagelin: Design and planning of the study; data collection, or data analysis and interpretation; statistical analysis; drafting and editing of the manuscript or critical review of important intellectual content; collection, analysis, and interpretation of data; effective participation in research orientation; intellectual participation in the propaedeutic and/or therapeutic conduct of the studied cases; critical review of the literature; approval of the final version of the manuscript.

## Conflicts of interest

None declared.
